# The Impact of Mutational Hotspots on Cancer Survival

**DOI:** 10.3390/cancers16051072

**Published:** 2024-03-06

**Authors:** Melissa Gonzalez-Cárdenas, Víctor Treviño

**Affiliations:** 1 Tecnologico de Monterrey, Escuela de Medicina y Ciencias de la Salud, Ave. Morones Prieto 3000, Monterrey 64710, Nuevo León, Mexico; melissagzz95@gmail.com; 2 Tecnologico de Monterrey, The Institute for Obesity Research, Eugenio Garza Sada Avenue 2501, Monterrey 64849, Nuevo León, Mexico; 3 Tecnologico de Monterrey, oriGen Project, Eugenio Garza Sada Avenue 2501, Monterrey 64849, Nuevo León, Mexico

**Keywords:** TCGA, recurrent mutations, log-rank, cox, VALORATE, biomarkers

## Abstract

**Simple Summary:**

In cancer, hotspots are those mutations emerging recurrently in tumors. Hotspots are highly likely to be functional because tumors tend to keep those mutations that provide physiological advantages. However, few hotspots have been studied, mainly because it is costly and time-consuming. Here, we systematically test more than 1400 hotspots for their association with patient survival and provide the results, including more than 300 significant associations, accessible on the web. This would help prioritize hotspots that are likely functional and affect patient survival, accelerating our knowledge of cancer and improving patient care.

**Abstract:**

Background: Cofactors, biomarkers, and the mutational status of genes such as TP53, EGFR, IDH1/2, or PIK3CA have been used for patient stratification. However, many genes exhibit recurrent mutational positions known as hotspots, specifically linked to varying degrees of survival outcomes. Nevertheless, few hotspots have been analyzed (e.g., TP53 and EGFR). Thus, many other genes and hotspots remain unexplored. Methods: We systematically screened over 1400 hotspots across 33 TCGA cancer types. We compared the patients carrying a hotspot against (i) all cases, (ii) gene-mutated cases, (iii) other mutated hotspots, or (iv) specific hotspots. Due to the limited number of samples in hotspots and the inherent group imbalance, besides Cox models and the log-rank test, we employed VALORATE to estimate their association with survival precisely. Results: We screened 1469 hotspots in 6451 comparisons, where 314 were associated with survival. Many are discussed and linked to the current literature. Our findings demonstrate associations between known hotspots and survival while also revealing more potential hotspots. To enhance accessibility and promote further investigation, all the Kaplan–Meier curves, the log-rank tests, Cox statistics, and VALORATE-estimated null distributions are accessible on our website. Conclusions: Our analysis revealed both known and putatively novel hotspots associated with survival, which can be used as biomarkers. Our web resource is a valuable tool for cancer research.

## 1. Introduction

Factors influencing cancer survival are crucial for patient stratification for therapies and the development of novel treatments. Several individual-level factors are commonly utilized, including age, sex, and ethnic group. Classical tumor factors such as TNM staging (size, nodal invasion, metastases), FIGO, or other tumor-specific classification systems also play a significant role. Pathology observations are also considered, such as the degree of differentiation or invasiveness. Molecular markers, single and multiple, have emerged as valuable tools for assessment. For instance, *ALK* rearrangements or overexpression in non-small-cell lung cancer [1] and the 70-gene signature known as Mammaprint [2] are used for prognosis. Moreover, many clinically informative molecular markers are associated with specific tumor mutations, such as *EGFR* in lung cancer [3], *BRCA1/2* in breast and ovarian cancers [4], or *BRAF* in melanoma [5]. Somatic molecular markers are currently preferred due to their higher accuracy in stratifying patients, ultimately improving overall survival rates.

Numerous genes exhibit recurrent mutations at specific positions across multiple patients, a phenomenon extensively identified through various computational methods [6]. These highly recurrent positions are commonly referred to as “hotspots”. We focus on hotspots at the nucleotide level in coding regions that have an impact on the translated protein, for example, a change in the amino acid at position 41, which is observed in several patients. Given the low likelihood of positional recurrence across diverse patients, hotspots are believed to result from positive selection during tumor progression, rendering them biologically and clinically significant [7]. Recently, there has been a growing interest in utilizing hotspots as survival markers due to their apparent associations with patient survival and their presumed distinct biological functions. However, investigating hotspots in this context has been limited to specific cases. Notably, hotspots within *TP53* have been extensively studied [8,9,10], revealing associations with survival in ovarian [9] and liver cancers [8]. Additionally, other hotspots have also been studied in specific cancers, such as *IDH1* and *IDH2* in gliomas [11], *EGFR* in the lung [3], and *BRAF* in skin cancer [12]. Throughout this manuscript, we explore more than 1400 potential hotspots from TCGA data and their potential associations with patient survival across diverse cancer types. 

**Figure 1:**
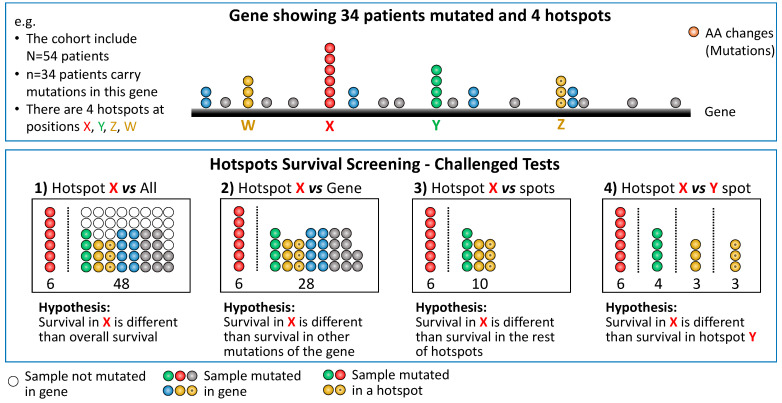
The four tests were performed to identify hotspots associated with survival. Top: as an example, a hypothetical gene is schematized for 54 patients, where 34 patients show mutations in their tumor sample. Each colored sphere corresponds to a patient carrying a mutation. An empty sphere corresponds to a patient not mutated in the gene. For this figure, 3 mutations in the same amino acid position defined a hotspot. Thus, there are 4 hotspots at positions X, Y, Z, and W. Bottom: the four tests performed. A vertical dashed bar splits the set of patients being tested. The four tests differ in the set of patients being compared with X, a specific hotspot (in red).

To discern the distinct clinical effects of hotspots, a commonly adopted practice involves comparing the survival of patients carrying mutations in a hotspot with those who do not [8]. This approach assumes that the survival of patients with the hotspot mutation differs from the overall survival rate. However, it is worth noting that the observed survival difference could be attributed to any other mutation in the gene rather than specifically to the hotspot. Therefore, an alternative and reasonable approach is to compare patients carrying the mutations in the hotspot with those carrying any other mutation in the same gene. An alternative and reasonable approach to identify differences in survival is by comparing a specific hotspot with another hotspot [9]. This comparison holds particular interest as it may aid in selecting hotspots for use in clinical settings, facilitating rapid tests. In this manuscript, we adopt a generalized approach to explore broader potential associations using four tests, as depicted in Figure 1.

We conducted a rigorous and systematic screening of 1469 candidate hotspots across 33 cancer types from the TCGA dataset. To ensure inclusivity and avoid specific criteria for hotspot selection, we focused on sequence-based hotspots, considering amino acid positions that exhibited mutations in four or more patients within a particular cancer type. To encompass various comparative scenarios, we implemented four different statistical tests for screening purposes (as illustrated in Figure 1). Subsequently, we performed 6451 evaluations encompassing hotspots in all genes and cancer types. The results of each test are presented, with specific examples being highlighted and discussed in detail. Overall, our study reveals that numerous hotspots demonstrate significant associations with survival, thereby underscoring their potential relevance for clinical applications. These findings pave the way for the possible use of hotspots in personalized treatment approaches, ultimately contributing to improved patient outcomes. The results are available in our web site (http://bioinformatics.mx/SurvHotspots).

## 2. Methods

### 2.1. Cancer Data

Cancer data for this study were obtained from The Cancer Genome Atlas (TCGA) data portal (https://portal.gdc.cancer.gov, accessed on 10 June 2021). We included a total of thirty-three different cancer types in our analysis. The mutations were obtained from MAF files generated through exome sequencing. The data files were dated 30 September 2017 (#filedate 20170930) with #annotation.spec tag as gdc-1.0.1-public. To extract overall survival data, we utilized Clinical TSV files. Annotations available within the MAF files, such as a brief description of amino acid alterations, transcript information, and gene identifiers, were utilized. We filtered mutations to use only those annotated with the field HGVSp_Short, which summarizes the amino acid change and position. The position and transcript ID were used to pool mutations. Silent mutations were not removed due to possible functional effects [13,14]. All data processing was performed using the R programming language, with the *maftools* package 2.18.0 [15] from Bioconductor (https://bioconductor.org/packages/release/bioc/vignettes/maftools/inst/doc/maftools.html, accessed first on 10 June 2021). We used CRAN R (https://cran.r-project.org/, accessed on 10 June 2021) version 4.1.1 in Mac and Windows.

### 2.2. Detection of Hypermutated Samples

Some samples showing an exacerbated number of mutations were excluded from the study to ensure robust analyses. The tumor mutation burden (TMB) was calculated as the total count of single nucleotide variations per sample. For further investigation and similarly to other studies [16,17], we classified samples as hypermutated if their TMB exceeded 500 mutations and if their TMB ranked within the top 10% among all samples. Additionally, we considered samples as hypermutated if their TMB was higher than the median plus four times the median absolute deviations of the TMB distribution.

### 2.3. Statistical Analyses

As depicted in Figure 1, our study involved the execution of four tests, all of which required the comparison of two survival curves. Traditionally, the log-rank test is commonly utilized for such comparisons. However, this test internally employs a χ^2^ distribution, assuming large sample sizes and comparable numbers of samples in both groups. This assumption becomes problematic in our analyses as the number of samples carrying a hotspot is expected to be small compared to the entire set of samples within a given cancer type. Past studies have established that the log-rank test is inappropriate for the proposed analyses [17,18,19]. To address this issue, we employed our R package VALORATE 1.0-1 [17,19], which accurately and efficiently estimates the exact null distribution for specific comparisons of survival curves. Briefly, the algorithm implemented in this package was designed to quickly estimate the distribution of the log-rank, especially for heavily unbalanced groups, as this is the case for hotspots. In addition to the *p*-value estimated by VALORATE, we also included the *p*-values calculated using the classical log-rank test and the Cox proportional hazard model as implemented in the *survival* package 3.5-8 in R. This comprehensive approach enables a more robust and reliable assessment of the statistical significance of the observed associations in our analyses. 

### 2.4. Test Strategies

We pursued our investigation using four distinct strategies outlined in Figure 1. Specifically, we considered amino acid positions with at least four patients containing clinical data, resulting in 1469 potential hotspots. For the second test, “Hotspot X vs. Gene”, the universe of hotspots was reduced to 1226. This reduction occurred because the patients were mutated in the same gene as the hotspot being tested. In some cases, this subset did not reach the minimum requirement of 4 patients for testing against the hotspot. In the third test, “Hotspot vs. Other Hotspots”, the set of patients was further reduced to those carrying a different hotspot, making 594 hotspots available for comparison. Finally, for the fourth test, “Hotspot X vs. Hotspot Y”, we compared each combination of hotspots within a gene, resulting in a total of 3162 combinations. All the results of these analyses are provided as supplementary files and have been deposited on our website for access and reference.

## 3. Results

In our comprehensive screening, we rigorously examined the association of hotspots with survival through four distinct tests, as illustrated in Figure 1. To elucidate these tests briefly, we evaluated whether the survival of a hotspot differs in terms of (a) the overall survival of all samples in the given cancer type (referred to as “Hotspot X vs. All”), (b) the survival of other mutations present in the same gene as the hotspot (“Hotspot X vs. Gene”), (c) the survival of other hotspots within the same gene (“Hotspot X vs. Spots”), or (d) the survival of a specific hotspot compared to another hotspot within the same gene (“Hotspot X vs. Hotspot Y”). We employed the VALORATE method, recalculating hotspots as previously performed in our work for HotSpotsAnnotations [20]. Nevertheless, we also provided classical log-rank tests and Cox model estimations. Additionally, to ensure the robustness of our analysis, we limited our investigations to hotspots with a minimum of four mutations.

### 3.1. Hypermutated Samples Bias Hotspots Associated with Cancer Survival

Our initial observation revealed a bias in the number of hotspots associated with survival, primarily due to the presence of hypermutated samples (Supplementary Figure S1). This finding aligns with previous reports indicating that the detection of hotspots can be influenced by the presence of hypermutated samples [16,17]. To ensure the integrity of our analyses, we proceeded to challenge the hotspots after excluding the hypermutated samples from further investigations. Details of the hypermutated samples removed during this process are presented in Supplementary Figure S2.

### 3.2. Many Hotspots Are Potentially Associated with Cancer Survival

Throughout our analysis across all cancer types, we methodically screened a total of 1469 distinct hotspots, resulting in noteworthy survival associations (*p* ≤ 0.05) for the “*X vs. All*” test in 95 cases, the “*X vs. Gene*” test in 44 cases, the “*X vs. Spots*” test in 44 cases, and 146 combinations for the “*X vs. Y*” test (Table 1). Among the cancer types examined, uterine corpus (UCEC), skin (SKCM), and colon (COAD) displayed a substantial number of detections in the first three tests (*vs. All*, vs. *Gene*, *vs. Spots*). On the other hand, 11 cancer types did not show any significant associations in these tests. These cancer types include adrenocortical (ACC), cholangiocarcinoma (CHOL), lymphoma (DLBC), kidney chromophobe (KICH), kidney renal papillary cell carcinoma (KIRP), pheochromocytoma and paraganglioma (PCPG), prostate (PRAD), sarcoma (SARC), testicular (TGCT), thyroid (THCA), thymus (THYM), and uterine carcinosarcoma (UCS). In the following section, we will analyze the results of each test.

**Figure 2:**
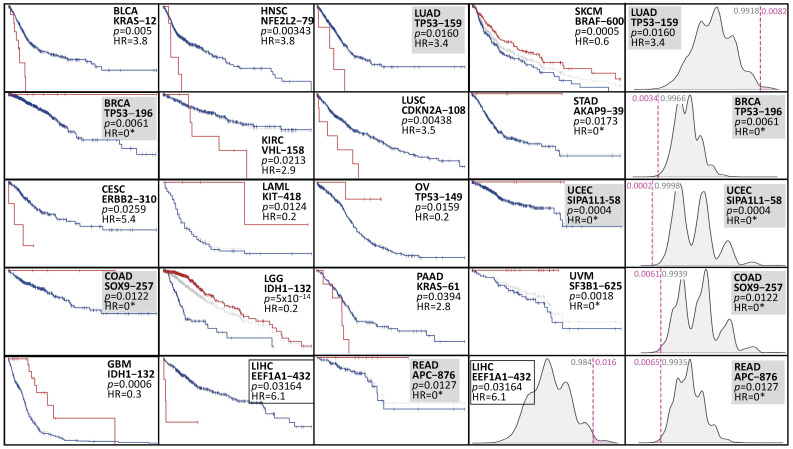
Survival curves represent the top significant hotspot for each cancer type. The red curve represent patients carrying the specified hotspot. The blue curve represent the rest of patients. The gray curve include all patients (for comparison). The axis of all curves has been removed to enhance clarity. Complete figures are accessible on our website. The non-symmetrical null distributions are depicted on the right, showcasing a selected example per row. In each row, the hotspot drawn on the right is highlighted with a gray square. The bottom row displays an additional distribution for *EEF1A1*, a gene that has received limited study in liver cancer. The *p*-values obtained from VALORATE are estimated by calculating twice the shaded pink area, corresponding to a two-sided test aimed at evaluating the log-rank statistic’s difference from zero, utilizing an empirical null distribution. * refers to underestimated HR given no events.

***Hotspot X vs. All***. When contrasting the survival of patients carrying a hotspot with those without carrying the hotspot, 95 associations were positive out of 1469 potential hotspots (Supplementary Table S1). Henceforth, in the subsequent text, we will refer to these 95 hotspots as being associated with survival. Among the 33 cancer types studied, 19 displayed at least one significant hotspot, with the most noteworthy hotspot for each cancer type shown in Figure 2. Intriguingly, some of these significant hotspots correspond to lesser-known genes or hotspots. For instance, *EEF1A1* observed in LICH is an elongation factor that has been demonstrated to play a critical role in efficient protein translation through phosphorylation at Ser300 [21], extending to other phosphorylation sites, including Thr432 [22]. Although overexpression of EEF1A1 in the liver enhances tumor progression [23], the mechanism behind the high-risk (hazard ratio = 3.8, *p* = 0.002) EEF1A1-432 hotspot in liver hepatocellular carcinoma, or any other cancer, remains unexplored. Similarly, NFE2L2, a leucine zipper transcription factor involved in antioxidant responses, has recently been implicated in head and neck radioresistance, particularly when carrying a mutation at amino acid 79 [24]. However, the mechanism underlying the hotspot NFE2L2-79 (HR = 3.8, *p* = 0.003) observed in HNSC (head and neck squamous cell carcinoma) remains poorly understood.

Except for IDH1-132, which is well-known in LGG and GBM [11], no other hotspot demonstrated significance in more than one cancer type, indicating that hotspots associated with survival in the “*vs. All*” test are specific to each cancer type. At the gene level, seven genes exhibited more than two significant hotspots or cancer associations (*TP53*, *KRAS*, *APC*, *IDH1*, *EGFR*, *CIC*, and *ARID1A*). Among them, *TP53* displayed 14 hotspot associations across nine different cancer types, with 3 observed in ovarian cancer and 2 in GBM, HNSC, and LUSC. Notably, five of these associations were associated with a lower risk, while nine were linked to a higher risk. *KRAS* displayed two hotspots in pancreatic cancer, including the well-known KRAS-12 [25] and KRAS-61. Additionally, KRAS-12 was observed in bladder cancer, and KRAS-13 was identified in uterine corpus (UCEC). These associations corresponded to a higher risk in the bladder and pancreas and a lower risk in UCEC. The gene APC also demonstrated three hotspot associations in closely related cancers, colon and rectal, all of which were associated with a lower risk. These associations suggest that the risk group depends on the specific gene and the context of the cancer type. EGFR, IDH1, ARID1A, and CIC also displayed two associations each, while the remaining 68 hotspots corresponded to one gene associated with one specific cancer type.

Table 2 presents the most frequently mutated hotspots observed in this analysis. At the top of the list is IDH1-132 in LGG, detected in 384 samples. Next, BRAF-600 in SKCM was observed in 141 samples, followed by KRAS-12 in PAAD, identified in 128 samples; GNAQ-209 in UVM, found in 37 samples; FGFR3-249 in BLCA, seen in 29 samples; PPP2R1A-179 in UCEC, present in 26 samples; AKT1-17 in BRCA, noted in 24 samples; EGFR-858 in LUAD, seen in 21 samples; and IDH2-172 in LGG, identified in 20 samples. All of these hotspots are well-known in cancer research and have been studied [3,11,12,26,27,28,29]. Interestingly, *PIK3CA* was the second most frequently tested gene across cancers, with 59 tests (followed by *TP53* with 235 tests). However, only the PIK3CA-1047 hotspot in COAD (*n* = 16) passed our threshold for significance. The functional relevance of this hotspot is well-established in the literature [30]. Recent studies have also shown the functional impact of PPP2R1A-179 in UCEC (*n* = 26) [27,31] and CIC-215 in glioblastomas [32]. Other less frequent but known hotspots include VHL-158 in KIRC (*n* = 7) [33], PPP6C-264 in SKCM (*n* = 7) [34], and SF3B1-625 in UVM (*n* = 13) [35]. The hotspot RUNX1-96 in BRCA, which is formed by frameshifts and lacks death events, has not been thoroughly studied. Still, overall, *RUNX1* mutations are well-known in leukemias [36], suggesting that this hotspot may have functional effects that deserve further investigation. Several other hotspots have not been well studied, such as those in *SLC3A2*, *OR14K1*, *SETD1B*, and *PTCH1*. 

Figure 2 also presents selected examples of multimodal null distributions arising from highly unbalanced groups and small sample sizes. These conditions violate the assumptions of the classical log-rank test, emphasizing the need for a more specific and robust statistical test, such as VALORATE.

***Hotspot X vs. Gene***. When comparing the survival of patients carrying a hotspot against all other patients with mutations in the gene, we identified significant hotspots in only 13 out of the 33 cancer types, resulting in a total of 44 associations from 1226 valid comparisons (Supplementary Table S2). The subsequent text will refer to this set of 44 hotspots associated with survival. Notably, no hotspot demonstrated significance in two or more cancer types, further suggesting specific associations between hotspots and particular cancer types. Only three genes (*APC*, *EGFR*, and *TP53*) exhibited two or more hotspots at the gene level. *APC* hotspots (−876 and −1489) were associated with a lower risk. In *EGFR*, two hotspots (−289 and −746) were linked to a lower risk in LGG and LUAD, respectively. EGFR-858 (n = 21) demonstrated marginal significance at *p* = 0.07, associated with a higher risk in LUAD. Among the 13 TP53 hotspots, ten were associated with a higher risk, with three (−176, −280, −126) being linked to LUSC (lung squamous cell carcinoma).

Due to the inherent similarity in the patient sets used for the analysis, it is unsurprising that several hotspots were also detected in the “X vs. All” test. For instance, hotspots such as BRAF−600, PPP2R1A−179, and AKT1−17 were also identified in the “X vs. All” test. This overlap in significant hotspots between the two tests reinforces the consistency and validity of our findings, indicating that these particular hotspots play critical roles in determining survival outcomes across different analyses.

To support the association of hotspots with survival, we present a brief description of biological and clinical findings related to the detected hotspots, focusing on those that were significant in the “X vs. Gene” test but not in the “X vs. All” test or were frequently mutated (Table 2). EGFR−746 in LUAD (*n* = 15) is a well-known hotspot [37] associated with a lower risk (HR = 0.22). In contrast, EGFR−858 showed marginal significance (*p* = 0.07) in LUAD, indicating a tendency for higher risk. The UPF3A−267 hotspot (*n* = 12) in UCEC has not been extensively studied, but UPF3A is known to inhibit the non-sense mediated decay response, implicated in more aggressive metastasis [38]. Our results are consistent with these findings, as UPF3A−267 (containing frameshifts) appeared to be more aggressive than other mutations in the gene (*n* = 6, no events). Regarding BMPR2−583, a recent study found *BMPR2* germline mutations in unexplained colon cancer cases, including the BMPR2−583 hotspot [39]. Our analysis observed that BMPR2−583 appeared to be more aggressive, although this estimation was based on only two events. *ATF7IP* encodes a protein that binds to the histone methyltransferase SETD1B, affecting immunogenicity when ATF7IP is depleted [40]. Our study found that ATF7IP−320 seemed more aggressive in UCEC, but this calculation was based on only two events. PSME4 is an essential component of the proteasome, modulating its activity and diversity in antigen presentation in lung cancer [41]. We observed the PSME4−1805 hotspot in STAD (*n* = 6, three events, median survival (MS) > 1000 days) compared with other mutations (*n* = 7, four events, MS = 400 days), suggesting that PSME4−1805 may behave differently. *CDKN2A* has multiple hotspots [42] and has been associated with poor survival in lung cancer [43]. The unstudied higher-risk CDKN2A−108 hotspot in LUSC (*n* = 6, all deaths) is the only hotspot significantly associated with survival in any cancer for this gene. The KAT6B−1203 hotspot detected in LGG (*n* = 6) has not been extensively studied, but the gene has been recently associated with modulating ferroptosis in gliomas [44]. In our analysis, we observed three deaths vs. zero deaths, suggesting that the mutations in 1203 may confer more aggressive characteristics.

***Hotspot X vs. Other Hotspots.*** Under the assumption that only recurrent mutation sites within the same gene are considered, which can be useful in clinical settings to select competing hotspots, we found 29 significant comparisons out of 594. These significant comparisons were distributed across 11 cancer types, with 5 in UCEC; 4 in LUSC and HNSC; 3 in OV, COAD, and LUAD; 2 in SKCM and LGG; and 1 in BRCA, GBM, and READ. The top hotspots, each with five or more samples, are presented in Supplementary Table S3. To summarize, let us discuss some of the genes or hotspots not present in the previous tests (Table 2). CTNNB1−37 has already been observed in cancers [45]. Our analysis showed that this hotspot in UCEC was associated with a higher risk for overall survival, a finding consistent with a recent study on endometrial carcinoma in Spain, which associated CTNNB1−37 with a higher risk for disease-free survival but not for overall survival on mutations in exon 3 [46]. This difference suggests that CTNNB1−37 behaves differently than other mutations in exon 3. *NFE2L2*, a transcription factor involved in the antioxidant response, exhibited interesting hotspots at position 29 in LUSC (*n* = 14) and HNSC (*n* = 4) and at position 79 in HNSC (*n* = 5). NFE2L2−79 has been linked to radioresistance in HNSC [24], and NFE2L2−29 and others have also been associated with radioresistance in LUSC [47]. However, the underlying mechanisms remain unknown. ARID1A-1989, observed in UCEC (*n* = 5), has been suggested to be involved in altering the epigenetic and immune response through interaction with EZH2 [48]. 

**Figure 3:**
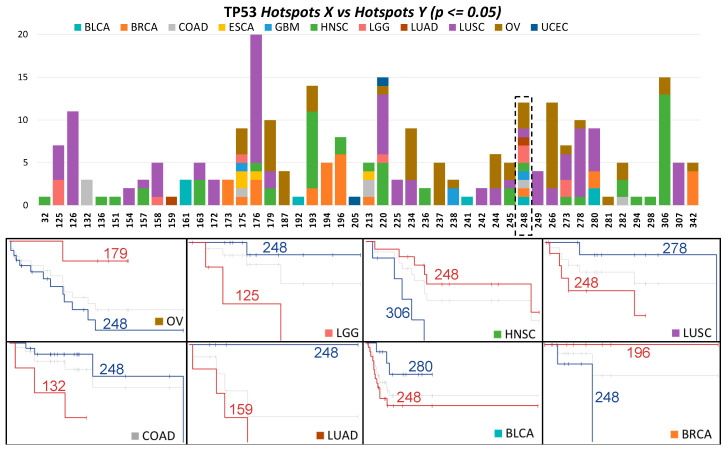
Hotspots in TP53 associated with survival across cancers in the *X vs. Y* comparison. The top bars display the hotspots per position and cancer type. The bottom Kaplan–Meier curves show examples of the hotspots at amino acid position 248 across eight of the nine observed cancer types.

***Hotspot X vs. Hotspot Y.*** In this part of the study, we compared specific hotspots within a gene, similar to previous work on TP53 [9]. Out of 3162 comparisons involving four or more mutations, we identified 146 significant associations at our screening threshold. Most of these significant associations (*n* = 132, 90%) were found in the TP53 gene, where we observed 47 significant hotspot positions across 11 cancer types (Figure 3). As an example, Figure 3 illustrates comparisons for the hotspot at position 248 in eight cancer types, with four showing a higher risk and four showing a lower risk. Similarly, the most recurrent significant hotspot was TP53−176 (observed in 20 out of 144 comparisons), showing higher risk in most comparisons. The next significant hotspot, TP53−306, was found in 18 comparisons, with higher risk observed in HNSC (*n* = 13) and OV (*n* = 2). Detailed information for many other comparisons is available on our website and in Supplementary Table S4. Apart from TP53, we identified 15 hotspot comparisons involving other genes (EGFR, APC, TTN, PIK3CA, CIC, ARID1A, FBXW7, and NFE2L2), presented in Table 2. For instance, PIK3CA−38 has been shown to be oncogenic [49]. In our comparisons, it displayed higher risk compared to PIK3CA−545 and PIK3CA−542. Similarly, PIK3CA−118 showed higher risk than PIK3CA−93 and PIK3CA−111 in UCEC. In LUAD, EGFR−858 is a well-known hotspot [3]. We found that the most relevant comparison was against EGFR−746, which showed a lower risk than EGFR−858 in LUAD. ARID1A−1850 is frequent in colorectal cancer [50]. In our study, among UCEC patients, 17 showed this mutation, and three death events were observed, compared to no events in ARID1A−1989. In colorectal cancer, APC exhibits various hotspots [51], including APC−213 and APC−876. In our comparisons, APC−213 showed higher risk than APC−876 and APC−1114, although only two events were registered.

### 3.3. Web Resource

**Figure 4:**
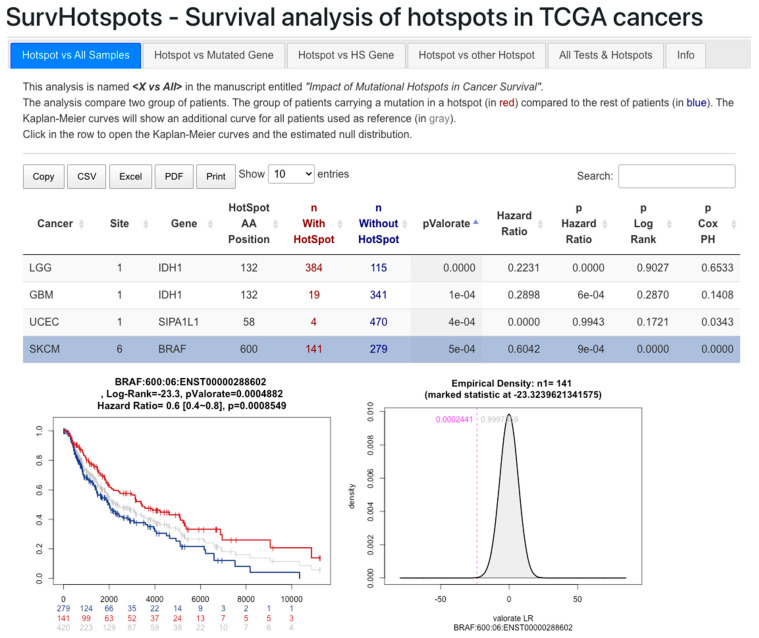
Preview (picture) of the web resource at http://bioinformatics.mx/SurvHotspots.

We have deposited the results of the four tests in the Supplementary Materials. Additionally, the Kaplan–Meier curves and the null distribution plots are available on our website at http://bioinformatics.mx/SurvHotspots. Figure 4 provides an example of a selected hotspot in the Hotspot X vs. All test. The results are presented by test, and all tests are also merged. They can be filtered by field and exported to common formats for further analysis and exploration.

## 4. Discussion

Hotspots hold significant biological and clinical relevance as they are believed to confer functional advantages in tumor progression [7]. Previous studies have demonstrated the impact of specific hotspots, such as those in *TP53*, *IDH1*, *EGFR*, and *BRAF*, among others, on survival in certain cancer cases [3,8,9,10,11,12]. To the best of our knowledge, our study represents the first systematic effort to comprehensively characterize hotspots across diverse cancer types. In this endeavor, we applied four approaches to explore potential survival associations among various cancer types. Through 6451 comparisons, we identified 314 associations with *p* < 0.05, encompassing well-known hotspots and others that have not been extensively studied. Detailed results are available in the Supplementary Materials and on our website.

This manuscript incorporates supporting information for functionally tested hotspots, revealing a mix of studied and unstudied hotspots within genes showing two or more hotspots. While some hotspots, such as EGFR−858 and EGFR−746, have been thoroughly investigated [3,37], other hotspots within the same gene, like EGFR−252 and EGFR−289 (*p* < 0.05 in LGG), remain unstudied. This pattern is consistent across various genes, highlighting the need for further investigations into unexplored hotspots. For instance, KRAS−12 and −13 have been well-studied [25], but KRAS−61 (*p* < 0.05 in PAAD) lacks similar attention. Similarly, although ARID1A−1989 is reported [48], ARID1A−1335 (*p* < 0.05 in UCEC) awaits functional assays. Likewise, SF3B1−625 is known [35], while SF3B1−902 (*p* = 0.066 in BLCA) has not been experimentally explored. TP53 is an extreme case with 64 hotspots, but only a few have been studied. Numerous other interesting hotspots have not been thoroughly investigated. Genes like TTK show potential as significant targets reported in breast, bladder, and prostate cancers [52,53,54,55], warranting further investigation of TTK−192 (*p* = 0.04 in UCEC) as an intriguing hotspot. Moreover, several other interesting hotspots accumulate numerous mutations without corresponding assays. For instance, genes *PPP2R1A*, *SLC3A2*, *OR14K1*, and *UPF3A* have over 10 mutations each, while *ATF7IP*, *PPP6C*, *ZFP37*, *PTCH1*, *DOCK3*, *ZNF728*, *PSME4*, *WNT1*, *NFRKB*, *PRSS35*, *KAT6B*, *ZMYND8*, and many others have 6 or more mutations. These hotspots could serve as potential targets for future studies. 

At the gene level, there are 18 genes showing more than five hotspots across all cancer types (in order, *TP53*, *PIK3CA*, *PTEN*, *APC*, *KRAS*, *CTNNB1*, *FBXW7*, *CDKN2A*, *ARID1A*, *PIK3R1*, *NFE2L2*, *EGFR*, *NRAS*, *BRAF*, *HRAS*, *VHL*, *IDH1*, *ERBB2*). From these, only four genes did not show a significant survival-associated hotspot (*PTEN*, *PIK3R1*, *NRAS*, *HRAS*). Nevertheless, one hotspot in *PTEN* and *NRAS* could be interesting (for PTEN-267, *p* = 0.06 at higher risk in STAD, *n* = 5, while for NRAS−61 *p* = 0.07 at lower risk in SKCM, *n* = 86 vs. 8 not mutated). Thus, most frequently “hotspoted” genes show survival associations.

Mutations in certain large genes, such as *TTN*, *NEB*, *SYNE1*, *MUC16*, and *OBSCN*, have previously been associated with tumor mutation burden (TMB) [56]. Among these genes, *NEB*, *SYNE1*, and *OBSCN* showed no recurrent amino acid (AA) position with four or more mutations, suggesting randomness. MUC16 exhibited five mutations in AA positions 5119 and 11,642 in SKCM and UCEC, respectively, but all comparisons had *p*-values greater than 0.05. On the other hand, *TTN* showed five AA positions with four or more mutations, and among these, TTN-20780 in SKCM displayed a significant association (*p* < 0.05) linked to lower risk. Interestingly, when a Cox proportional hazard model of the SKCM data (type 06) was fitted to TMB (in logarithm scale), it yielded a significant result (*p* = 0.000842) associated with lower risk. This finding suggests that TTN−20780 or a broader region may be a potential surrogate marker for TMB in SKCM.

Interestingly, we observed that the majority of hotspots exhibited cancer-type-specific associations, underscoring the significance of considering both the hotspot and the cancer-type context. These findings highlight the intricate interplay between genetic alterations and the underlying biology of various cancer types, emphasizing the necessity for tailored therapeutic approaches based on the specific genetic landscape of each patient’s tumor.

Indeed, it is important to acknowledge the limitations and complexities associated with testing hotspots for an association with survival independently of other factors. In many studies, including this one, accounting for all potential confounding factors, such as treatment, age at diagnosis, sex, cancer heterogeneity, and molecular subtypes that can influence survival times, presents a challenge. These factors may vary significantly among different cancer types, making a systematic analysis considering all these variables difficult. Furthermore, in cases where the number of samples is limited, as observed in many hotspots, introducing a correcting factor in statistical models can complicate the estimation of possible effects and may lead to unstable or biased results. Balancing the need for correction with the risk of overfitting or introducing additional biases into the analysis is essential. Despite these challenges, the estimations provided in this study offer valuable insights into potential associations between hotspots and survival outcomes. By highlighting hotspots that show a significant association with survival, this research can serve as a basis for further investigation and validation of these findings. Additionally, identifying hotspots likely to have functional effects on tumor progression can inform future research and therapeutic strategies. Researchers must be aware of these limitations and interpret the results in the context of the study design and the data available.

Indeed, using a raw *p*-value threshold of 0.05 is a common approach for filtering significant associations in statistical analyses. However, it is essential to recognize that some associations may still be of potential interest, even if they do not reach strict statistical significance (*p* < 0.05). Marginal associations, such as those with *p*-values close to 0.05, can still provide valuable insights and warrant further investigation. For example, the GNAQ-209 hotspot, despite not meeting the strict significance threshold, may still be biologically and clinically relevant, especially given its association with lower risk. This finding aligns with previous research on this hotspot in uveal melanoma [57,58], and its potential implications in tumor progression and patient outcomes could be worth exploring in more detail. Similarly, the ATAD2B−307 hotspot, despite having limited studies, may represent an interesting candidate for further investigation. The fact that it shows an association with higher risk (albeit marginally significant) suggests that it could be involved in tumor aggressiveness or disease progression. Given its role in interacting with histones [59], studying the functional implications of this hotspot could shed light on its potential role in cancer development. Overall, while a strict *p*-value threshold is a useful way to identify statistically significant associations, it is crucial not to overlook potentially important findings that fall just below this threshold. Researchers should consider such marginal associations in the context of the existing literature, biological plausibility, and potential clinical implications. Further functional studies and validation in independent datasets can help confirm the significance of these marginal associations and their relevance in cancer biology.

In our study, we conducted numerous statistical tests to assess the association of hotspots with survival across various cancer types. This procedure presents the challenge of multiple testing and the potential for increased false positive calls. While there are several methods to address with multiple testing approaches [60], it is recognized that some approaches may overcorrect *p*-values [61]. To address this concern, we adopted a data-driven approach to estimate the null distribution on a per-case basis, taking into account specific conditions of each test, such as the total number of patients and the number of patients carrying a hotspot in each cancer type. By tailoring the correction to the individual characteristics of each test, our goal was to mitigate the risk of overcorrection and provide a more accurate assessment of statistical significance. This approach acknowledges that the assumptions of traditional *p*-value correction methods, which assume a single common null distribution, may not hold in our context. However, it is crucial to acknowledge that despite our efforts to address multiplicity, caution is still warranted, especially when dealing with hotspots having a low number of mutations. In some cases, the sample size for certain hotspots or cancer types may be limited, impacting the reliability of statistical estimates. To account for these limitations, we transparently present the results, including *p*-values, hazard ratios, and null distributions, enabling researchers to assess the robustness of the associations. Moreover, we advise prioritizing hotspots for further validation based on additional criteria, such as biological relevance, existing supporting evidence, and potential clinical implications.

In this study, we opted to use the VALORATE R package, a tool we previously proposed for analyzing survival data with small sample sizes and unbalanced groups. The classical log-rank test, which assumes large sample sizes and similar group sizes, can pose challenges when applied to situations with small samples. To illustrate the limitations of using the log-rank test in such scenarios, we present two examples. In the X vs. Y test, comparing hotspot TP53−175 vs. TP53−273 in STAD, the log-rank test yielded a *p*-value of 0.019, while VALORATE produced a *p*-value of 0.299 (Supplementary Figure S3). This discrepancy underscores the impact of using a generic test that assumes large and balanced sample sizes when the data violate these assumptions. In this case, VALORATE’s estimation of the null distribution better accounts for the specific characteristics of the data, leading to a more reliable *p*-value. In the X vs. All test, the well-known hotspot IDH1-132 in gliomas demonstrated a clear difference in survival curves, with 384 patients carrying the mutation compared to 115 patients without it. The log-rank test estimated a *p*-value of 0.9027, while VALORATE’s computation resulted in a *p*-value approaching zero (*p* < 10^−13^, as shown in Supplementary Figure S4). The substantial difference in sample sizes among the groups in this case violates the log-rank test’s assumption, leading to an inaccurate *p*-value. Once again, VALORATE’s approach provides a more accurate and meaningful assessment of statistical significance. By relying on VALORATE’s more appropriate estimation of the null distribution, we aim to avoid false-positive or false-negative calls in our results, ensuring a more robust and reliable identification of hotspots associated with survival across diverse cancer types.

## 5. Conclusions

In conclusion, our comprehensive analysis of hotspots across a wide spectrum of cancer types sheds light on their potential role as survival biomarkers. The identified hotspots, encompassing both established and novel associations, constitute a valuable resource for future studies and may contribute to the development of personalized treatment strategies for cancer patients. Briefly, hotspots can be added as clinical information to inform higher or lower risk or to design new prognostic models. With the availability of our results in supplementary tables and on our website, we ensure accessibility and encourage further exploration by the scientific community. Subsequent research should delve into the functional implications of these hotspots and assess their potential as targets for therapeutic interventions, ultimately advancing patient outcomes in precision oncology.

## Figures and Tables

**Table 1:** Summary of results.

Cancer Type	Potential Hotspots	X vs. All	X vs. Gene	X vs. HS	X vs. Y
ACC	1	-	-	-	-
BLCA	50	2	-	-	4
BRCA	56	3	3	1	14
CESC	10	1	-	-	-
CHOL	1	-	-	-	-
COAD	108	10	6	3	4
DLBC	2	-	-	-	-
ESCA	15	-	-	-	2
GBM	23	3	1	1	2
HNSC	50	3	2	4	27
KICH	0	-	-	-	-
KIRC	7	1	1	-	-
KIRP	3	-	-	-	-
LAML	8	1	-	-	-
LGG	35	6	3	2	7
LIHC	15	1	-	-	-
LUAD	29	2	2	3	2
LUSC	49	3	4	4	45
MESO	0	-	-	-	-
OV	38	3	3	3	28
PAAD	13	2	-	-	-
PCPG	3	-	-	-	-
PRAD	8	-	-	-	-
READ	15	2	1	1	3
SARC	3	-	-	-	-
SKCM	178	18	6	2	1
STAD	95	2	2	-	-
TGCT	4	-	-	-	-
THCA	5	-	-	-	-
THYM	4	-	-	-	-
UCEC	631	31	10	5	7
UCS	7	-	-	-	-
UVM	3	1	-	-	-
**Sum**	**1469**	**95**	**44**	**29**	**146**
**Comparisons**		**1469**	**1226**	**594**	**3162**

**Table 2:** Selected top mutated hotspots associations.

Test	Cancer	Gene	Hotspot Position	n (with/without)	*p*	HR
*X* vs. *All*	LGG	*IDH1*	132	384/114	0	0.2
	SKCM *	*BRAF*	600	141/182	0.01	0.7
	PAAD	*KRAS*	12	128/48	0.04	1.6
	SKCM	*BRAF*	600	47/373	0.05	2.9
	UVM	*GNAQ*	209	37/42	0.053	0.4
	BLCA	*FGFR3*	249	29/357	0.04	0.5
	UCEC	*PPP2R1A*	179	26/448	0.04	2.4
	BRCA	*AKT1*	17	24/918	0.02	<1
	LUAD	*EGFR*	858	21/457	0.03	2
	LGG	*IDH2*	172	20/478	0.04	0.2
	GBM	*IDH1*	132	19/340	0	0.3
	UCEC	*SLC3A2*	300	17/457	0.03	<1
	COAD	*PIK3CA*	1047	16/326	0.05	0.2
	UCEC	*KRAS*	13	14/460	0.04	<1
	UVM	*SF3B1*	625	13/66	0	<1
	UCEC	*OR14K1*	14	13/461	0.01	<1
	LGG	*CIC*	215	12/486	0	<1
	GBM	*TP53*	248	12/347	0.01	0.5
	COAD	*SETD1B*	8	12/330	0.04	<1
	BLCA	*KRAS*	12	10/376	0	3.8
	LIHC	*TP53*	249	10/339	0.054	2.6
	HNSC	*TP53*	193	9/466	0.02	3.1
	BRCA	*TP53*	196	8/934	0.01	<1
	READ	*APC*	876	8/110	0.01	<1
	BRCA	*RUNX1*	96	7/935	0.04	<1
	UCEC	*PTCH1*	1203	7/467	0.03	<1
	UCEC	*ZFP37*	161	7/467	0.03	<1
	KIRC	*VHL*	158	7/323	0.02	2.9
	SKCM *	*PPP6C*	264	7/316	0.04	0.4
	PAAD	*KRAS*	61	7/166	0.04	2.8
*X* vs. *Gene*	LUAD	*EGFR*	746	15/43	0.01	0.2
	UCEC	*UPF3A*	267	12/7	0	>1
	COAD	*BMPR2*	583	9/8	0.04	>1
	UCEC	*ATF7IP*	320	7/14	0.01	>1
	COAD	*DOCK3*	1852	7/12	0.04	<1
	STAD	*PSME4*	1805	6/7	0.03	<1
	LUSC	*CDKN2A*	108	6/64	0.01	3.4
	LGG	*KAT6B*	1203	6/5	0.04	>1
	LUSC	*TP53*	126	6/361	0.05	4.5
	HNSC	*TP53*	306	6/310	0.02	4.5
	UCEC	*ZMYND8*	635	6/18	0.04	<1
	COAD	*FBXW7*	505	5/40	0.04	4.4
	SKCM *	*SALL1*	675	5/37	0.01	<1
	LUSC	*TP53*	176	5/362	0.01	4.1
	OV	*TP53*	179	5/359	0.02	0.2
	OV	*TP53*	244	5/359	0.03	0.2
	OV	*TP53*	266	5/359	0.02	3.2
*X* vs. *Other HS*	UCEC	*KRAS*	12	65/17	0.04	>1
	UCEC	*CTNNB1*	37	19/69	0.04	3.8
	LUSC	*NFE2L2*	29	14/35	0.05	2.8
	LGG	*TP53*	248	14/128	0.04	0.3
	UCEC	*PIK3CA*	38	10/172	0.03	3.9
	LUAD	*TP53*	125	10/144	0.01	3.9
	UCEC	*ARID1A*	1989	5/72	0.04	<1
	HNSC	*NFE2L2*	79	5/4	0.05	2.6
*X vs. Y*	UCEC	*ARID1A*	1850 vs. 1989	17/5	0.03	>1
	LUAD	*EGFR*	746 vs. 858	15/21	< 0.01	0.2
	UCEC	*FBXW7*	505 vs. 545	12/4	0.04	<1
	UCEC	*PIK3CA*	38 vs. 545	10/26	0.04	5.5
	UCEC	*PIK3CA*	38 vs. 542	10/22	0.03	9.1
	UCEC	*PIK3CA*	118 vs. 93	9/8	0.03	>1
	READ	*APC*	213 vs. 876	6/8	0.04	>1
	READ	*APC*	1114 vs. 213	5/6	0.02	<1
	READ	*APC*	1114 vs. 1450	5/4	0.05	<1
	UCEC	*PIK3CA*	111 vs. 118	4/9	0.04	<1
	HNSC	*NFE2L2*	29 vs. 79	4/5	0.05	<1
	LGG	*CIC*	201 vs. 215	4/12	0.03	>1
	LGG	*CIC*	202 vs. 215	4/12	0.04	>1

Only those whose total number of mutated samples is seven or more for the “X vs. All test” or five or more for the other tests. The complete list is included in the Supplementary Materials. * Mark TCGA type = 06, metastatic site samples. HR refers to the hazard ratio. HR marked “<1” or “>1” seems to be associated with low- or high-risk, respectively, with HR estimation not clear due to a low number of samples and/or lack of events in one sample.

## Data Availability

The data generated in this work are available at http://bioinformatics.mx/SurvHotspots.

## Supplementary Materials

The following supporting information can be downloaded at https://www.mdpi.com/article/10.3390/cancers16051072/s1. Figure S1. Detection of significant hotspots in UCEC before and after hypermutated sample removal; Figure S2. Tumor mutation burden (TMB) per cancer type and removed hypermutated samples; Figure S3. Comparison of hotspots TP53-175 (red) vs. TP53-273 (blue) in STAD cancer; Figure S4. Comparison of hotspots in IDH1 (red) vs. All other patients not carrying mutation (blue) in LGG; Table S1. Hotspots evaluated in the X vs. All Test; Table S2. Hotspots evaluated in the X vs. Gene Test; Table S3. Hotspots evaluated in the X vs. Other Hotspots Test; Table S4. Hotspots evaluated in the X vs. Y Test.
