# Supplementary material for: The Impact of Mutational Hotspots on Cancer Survival
Source: Cancers (Basel). 2024 Mar 6;16(5):1072. doi: 10.3390/cancers16051072 (PMC10931094; doi:10.3390/cancers16051072)
Supplement: Supplementary file 1 [file cancers-16-01072-s001.zip › supplementary Figures.pdf]

Supplementary Figures

The Impact of Mutational Hotspots in Cancer Survival

Melissa Gonzalez-Cárdenas [1,2] and Víctor Treviño [1,2,3,*]

[1] Tecnologico de Monterrey, Escuela de Medicina y Ciencias de la Salud, Ave. Morones Prieto 3000,
Monterrey 64710, Nuevo León, Mexico; melissagzz95@gmail.com
[2] Tecnologico de Monterrey, The Institute for Obesity Research, Eugenio Garza Sada Avenue 2501,
Monterrey 64849, Nuevo León, Mexico
[3] Tecnologico de Monterrey, oriGen Project, Eugenio Garza Sada Avenue 2501,
Monterrey 64849, Nuevo León, Mexico
* Correspondence: vtrevino@tec.mx

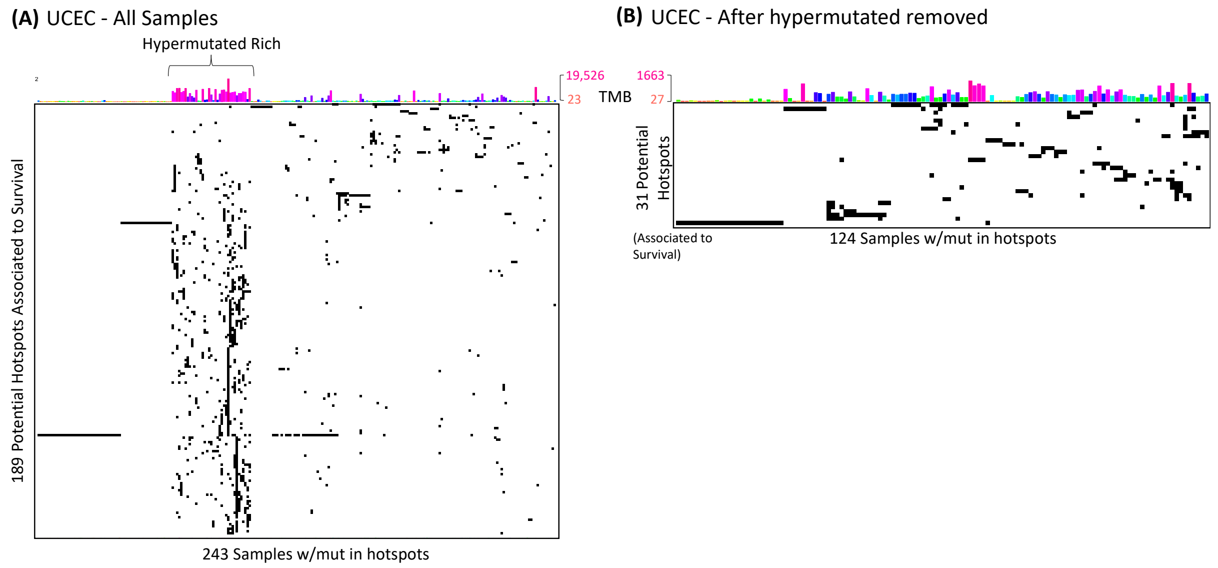

Supplementary Figure S1. Detection of significant hotspots in UCEC before and after hypermutated samples removal. (A) Using all samples in UCEC where the p-value < 0.05 yields 189 hotspots. Patients are shown in columns and hotspots in rows. Samples and hotspots are sorted by a hierarchical clustering algorithm. A black dot maps a patient carrying mutation in a hotspot. Upper bars show the total mutation burden (TMB). Note the region around the center that is rich in highly mutated samples. This may be indicative of bias in detection given TMB. (B) Using samples after removing 53 hypermutated samples in UCEC, the dependency of TMB and hotspots is not present. The samples removed per cancer type is shown in Supplementary Figure S2.

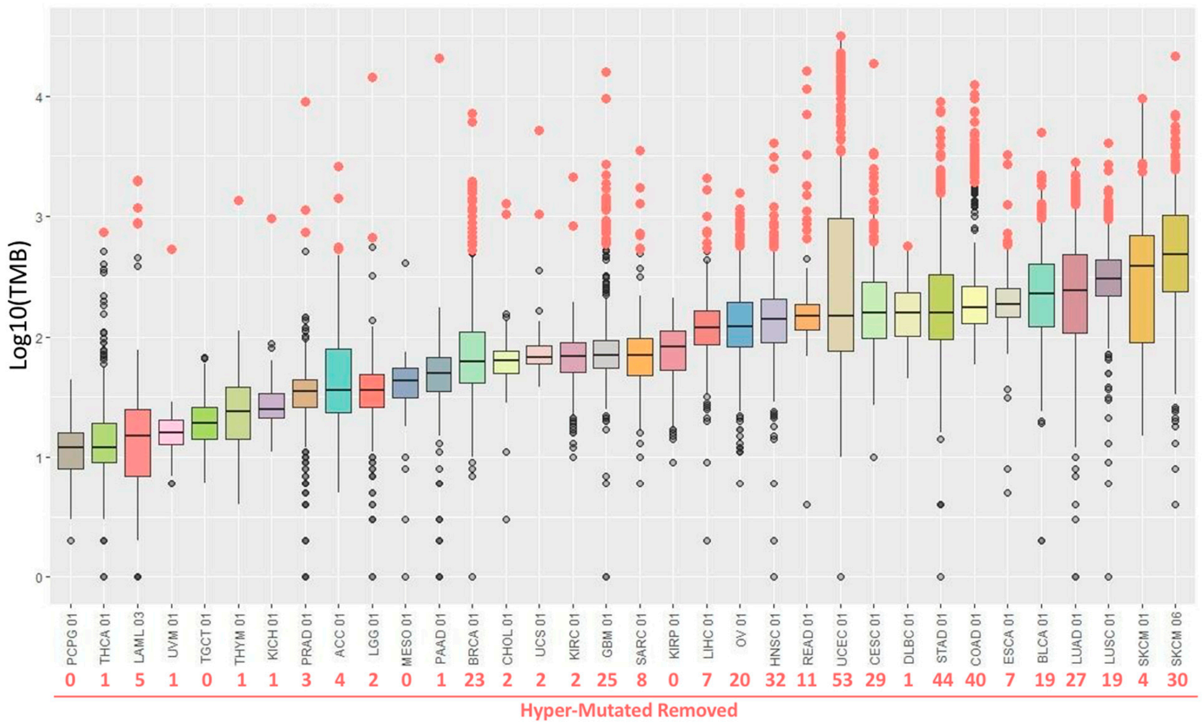

Supplementary Figure S2. Tumor mutation burden (TMB) per cancer type and removed hypermutated samples. Boxplots show the TMB per cancer type in logarithm base 10 scale. Outliers are shown. Samples were considered hypermutated if TMB is higher than 500 mutations, TMB is in the top 10%, and TMB is higher than median + 4 median absolute deviations of the TMB distribution. The number of hypermutated samples are shown at the bottom and as red dots.

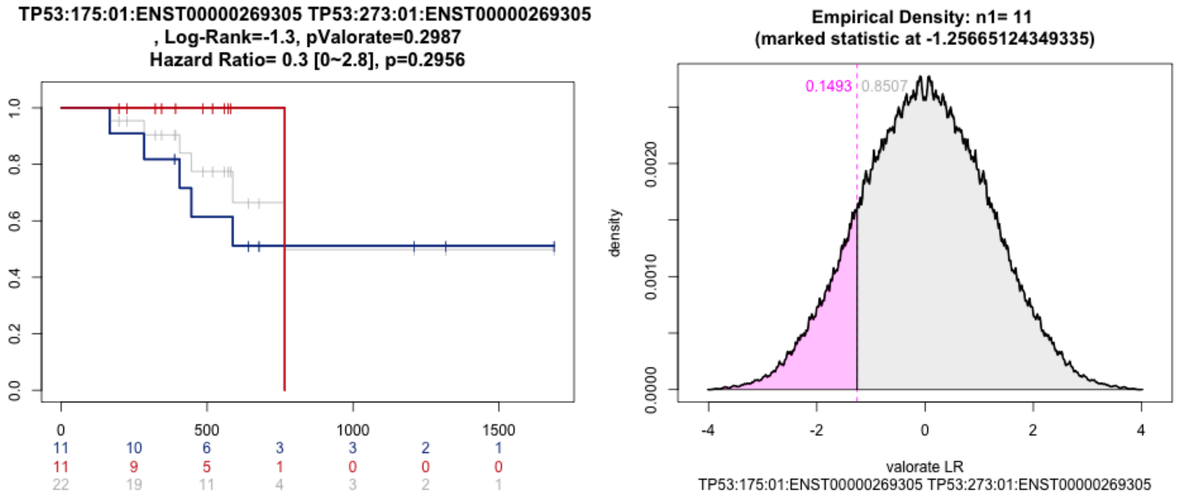

Supplementary Figure S3. Comparison of hotspots TP53-175 (red) vs. TP53-273 (blue) in STAD cancer. In the null distribution at the right, the peaky curve is an exact distribution instead of an approximation. This curve is computable because the total size is only 22 (it takes a couple of

minutes), which is built in VALORATE sampling all combinations instead of performing an approximation.

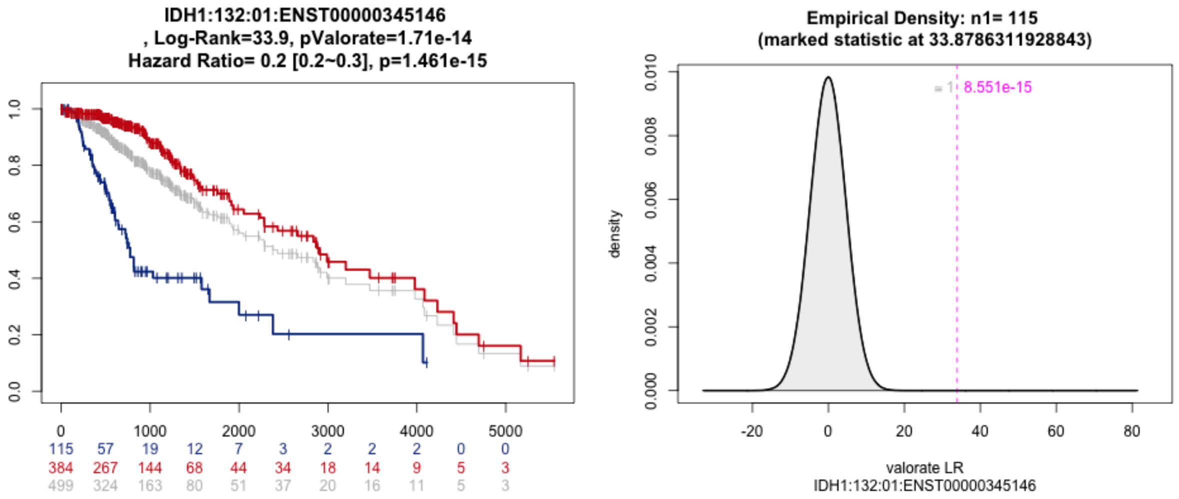

Supplementary Figure S4. Comparison of hotspot in IDH1 (red) vs. All other patients not carrying mutation (blue) in LGG. The gray Kaplan-Meier curve, representing all samples, is shown for comparison purposes only.
